# Analysis of widefield choroidal thickness maps of healthy eyes using swept source optical coherence tomography

**DOI:** 10.1038/s41598-023-38845-9

**Published:** 2023-07-24

**Authors:** Masatoshi Hirano, Yuki Muraoka, Takahiro Kogo, Masaharu Ishikura, Naomi Nishigori, Naoko Ueda-Arakawa, Manabu Miyata, Masayuki Hata, Ayako Takahashi, Masahiro Miyake, Akitaka Tsujikawa

**Affiliations:** Department of Ophthalmology and Visual Sciences, Kyoto University Graduate School of Medicine, Sakyo-ku, Kyoto, 606-8507 Japan

**Keywords:** Medical research, Characterization and analytical techniques

## Abstract

We aimed to obtain widefield (WF) swept source optical coherence tomography (SS-OCT) data and examine the features of choroidal thickness maps in healthy eyes. The posterior pole choroidal thickness was examined in 127 eyes using enhanced depth imaging of SS-OCT with a viewing angle of 20 (vertical) × 23 (horizontal) mm, and choroidal thickness maps were generated. For SS-OCT image analysis, we developed a grid with inner and outer rings, each divided into superotemporal, inferotemporal, superonasal, and inferonasal quadrants, comprising a total of nine subfields, including the central 3-mm ring. The posterior pole choroidal thicknesses were significantly lower at the periphery than in the central area, in the inferior field than in the superior field, and in the nasal field than in the temporal field (*p* < 0.001 for all). We also evaluated the effects of age and axial length (AL) on the WF choroidal thickness. The choroidal thickness in all subfields was negatively associated with advanced age (*p* < 0.05). The choroidal thicknesses in the central and inferonasal inner and outer subfields were negatively associated with AL (*p* = 0.042, 0.034, and 0.022, respectively). These findings provide insights into the two-dimensional characteristics of choroidal thickness and its association with age and AL.

## Introduction

The choroid, which forms the posterior uvea, is a melanin-rich membranous tissue with abundant vasculature and stroma. It lies between the retina and sclera and plays an important role in supplying oxygen and nutrients to the outer retina, regulating intraocular pressure, and absorbing excess light^1–5^. Considering these functions, the characteristics of the choroidal structure in eyes with pathologies, such as age-related macular degeneration (AMD), central serous chorioretinopathy (CSC), and high myopia, have been discussed previously^6–9^.

Highly sensitive and early detection of pathological changes in the choroid requires thorough knowledge of its anatomical features. Previous studies using optical coherence tomography (OCT) B-scans showed that the macular choroidal thickness was negatively associated with aging and an increase in the axial length (AL)^10,11^, and that the macular choroid was the thickest on the superior side, followed by the foveal area and the temporal, inferior, and nasal sides^12^.

Recent technological advances in OCT imaging, particularly enhanced-depth imaging (EDI) and swept-source (SS) OCT, have facilitated wide-field (WF) and quantitative evaluation of the choroidal structure^13,14^. Findings of recent studies using WF SS-OCT imaging of eyes with CSC showed choroidal thickening in the vicinity of the vortex vein ampulla along the course of the vertically and asymmetrically dilated choroidal veins, which was suggestive of the pathogenesis^15–17^. The collection of WF choroidal data from healthy eyes is pivotal for the precise evaluation of pathological alterations in the choroidal structures of eyes with retinal choroidal diseases, particularly during the initial stages. Several previous studies have investigated the choroid not only in the central fovea but also outside the macula, discussing its relationship with factors, such as age, AL, and sex^18,19^. However, most of these studies primarily featured small sample sizes or qualitative evaluations, resulting in a lack of unified consensus. Thus, further studies exploring the characteristics of the WF choroidal thickness in healthy eyes are required.

Accordingly, the aim of the present study was to obtain WF SS-OCT data and quantitatively evaluate choroidal thicknesses as well as differences in choroidal thicknesses between the superotemporal, inferotemporal, superonasal, and inferonasal quadrants and between the peripheral and macular regions in the healthy eyes of Japanese participants. We also examined the association of age and AL with peripheral choroidal thickness because these factors have been reported to be strongly associated with changes in macular choroidal thickness^12^.

## Results

**Figure 1:**
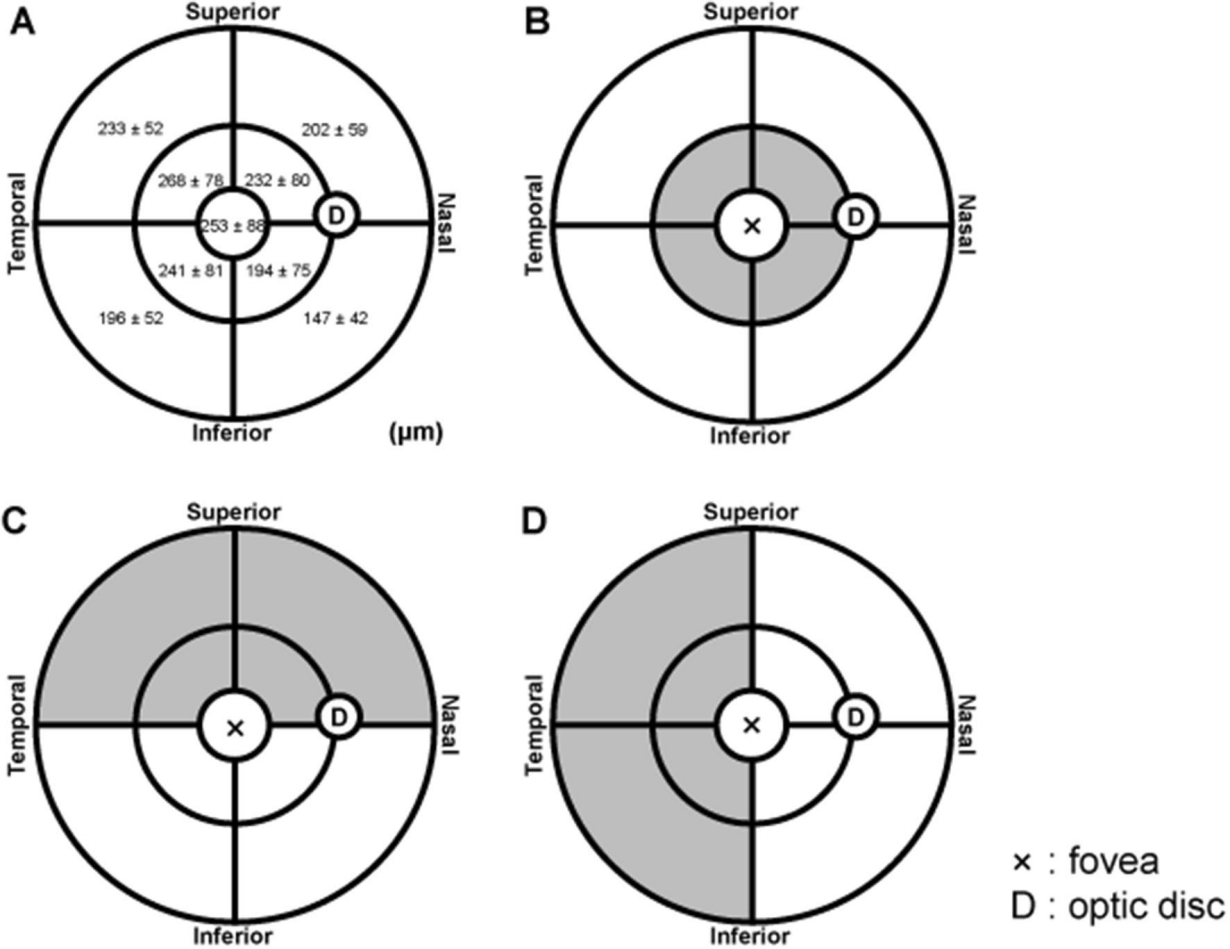
Choroidal thickness map analysis using enhanced-depth imaging of widefield swept source optical coherence tomography. (**A**) Mean choroidal thickness in each subfield. (**B**) Comparisons of widefield (WF) choroidal thicknesses between corresponding quadrants in the inner and outer rings. The choroidal thickness in the outer ring (white area) was significantly lower than that in the corresponding quadrants of the inner ring (gray area; *p* < 0.001 for all). (**C**) Comparisons of WF choroidal thicknesses between corresponding superior and inferior subfields. The choroidal thicknesses in the inferior subfields (white area) were significantly lower than those in the corresponding superior subfields (gray area; *p* < 0.001 for all). (**D**) Comparisons of WF choroidal thicknesses between corresponding temporal and nasal subfields. The choroidal thicknesses in the nasal subfields (white area) were significantly lower than those in the corresponding temporal subfields (gray area; *p* < 0.001 for all).

Table 1 shows the background characteristics of the 127 participants (58 men and 69 women). The mean age of the participants was 59.9 ± 17.3 years, and the mean AL was 24.19 ± 1.08 mm. Figure 1 shows the mean choroidal thickness in each examined subfield. For each participant, a clear choroidal thickness map was generated, in which the choroid at the posterior pole showed continuous thinning from the macula to the periphery. However, the area along the large choroidal vessels (vortex veins) was thicker than the surrounding area (Supplementary Fig. S1 online).

**Table 1:** Characteristics of the healthy eyes included in the study.

Number (men/women)	127 (58/69)
Age, years (range, years)	59.9 ± 17.3 (20–87)
Height, cm (range, cm)	161.4 ± 8.0 (146–178)
Weight, kg (range, kg)	61.6 ± 11.5 (40.9–96.1)
Axial length, mm (range, mm)	24.19 ± 1.08 (22.01–25.95)
Spherical equivalent, D (range, D)	−1.00 ± 2.20 (−5.88 to 3.00)
LogMAR visual acuity (range)	0.10 ± 0.01 (−0.18 to 0.22)
(range in Snellen equivalent)	(12/20–20/13)

The data are shown as mean ± standard deviation unless otherwise indicated.

*LogMAR* logarithm of minimum angle of resolution.

We compared the choroidal thicknesses between the corresponding subfields in the inner and outer rings, between the corresponding areas in the superior and inferior subfields, and between the corresponding areas in the temporal and nasal subfields (Fig. 1). Choroidal thickness was significantly lower in the outer ring subfields than in the corresponding inner ring subfields (*p* < 0.001), in the inferior subfields than in the corresponding superior subfields (*p* < 0.001), and in the nasal subfields than in the corresponding temporal subfields (*p* < 0.001 for all; Fig. 1).

**Figure 2:**
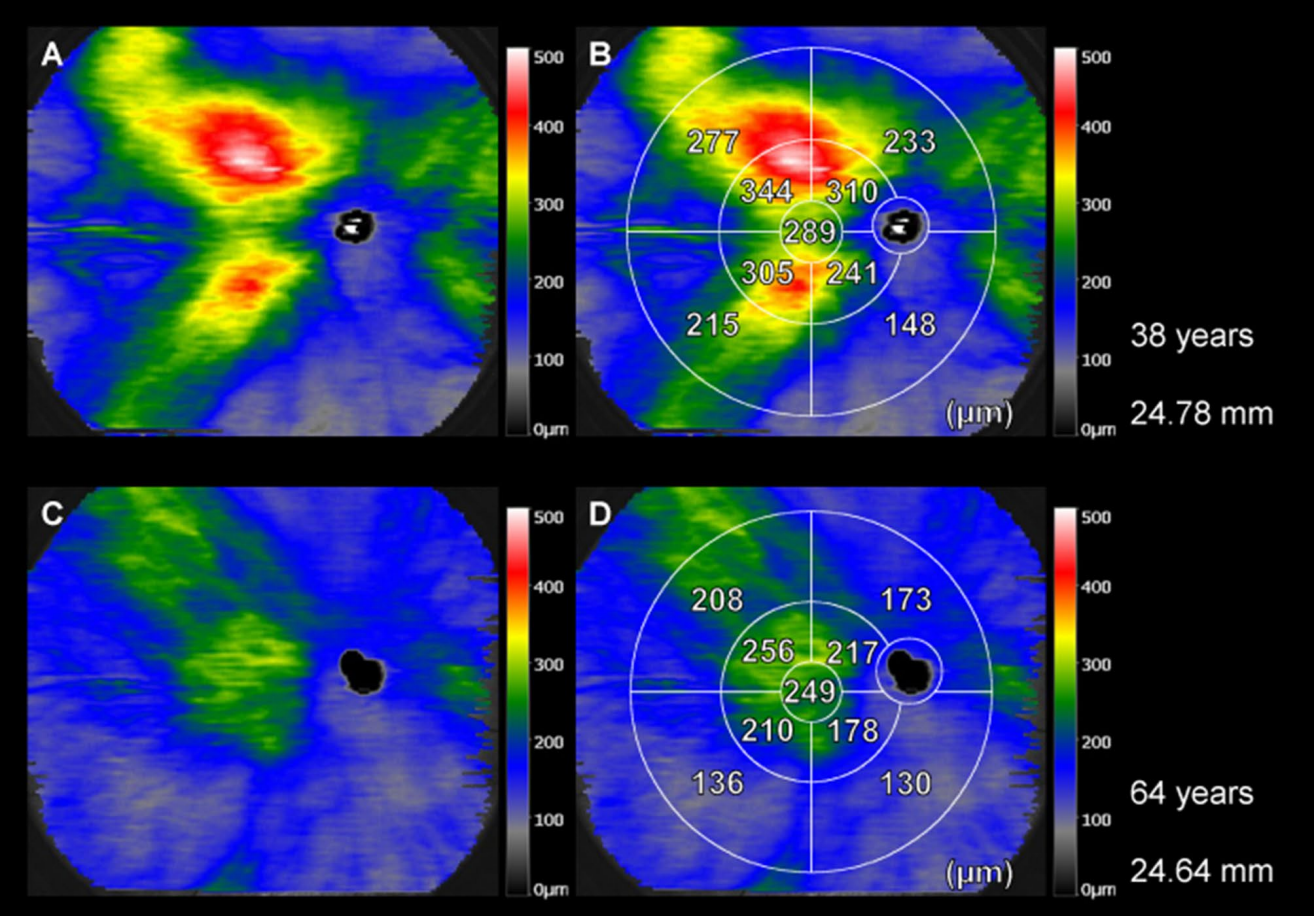
Differences in choroidal thickness with age. (**A,B**) A wide-field (WF) choroidal thickness map of a 38-year-old man. The mean axial length (AL) of the eye was 24.78 mm. (**C,D**) A WF choroidal thickness map of a 64-year-old man. The mean AL in the eye was 24.64 mm. These findings suggest that the choroid is thinner in older individuals than in young individuals in all subfields.

**Figure 3:**
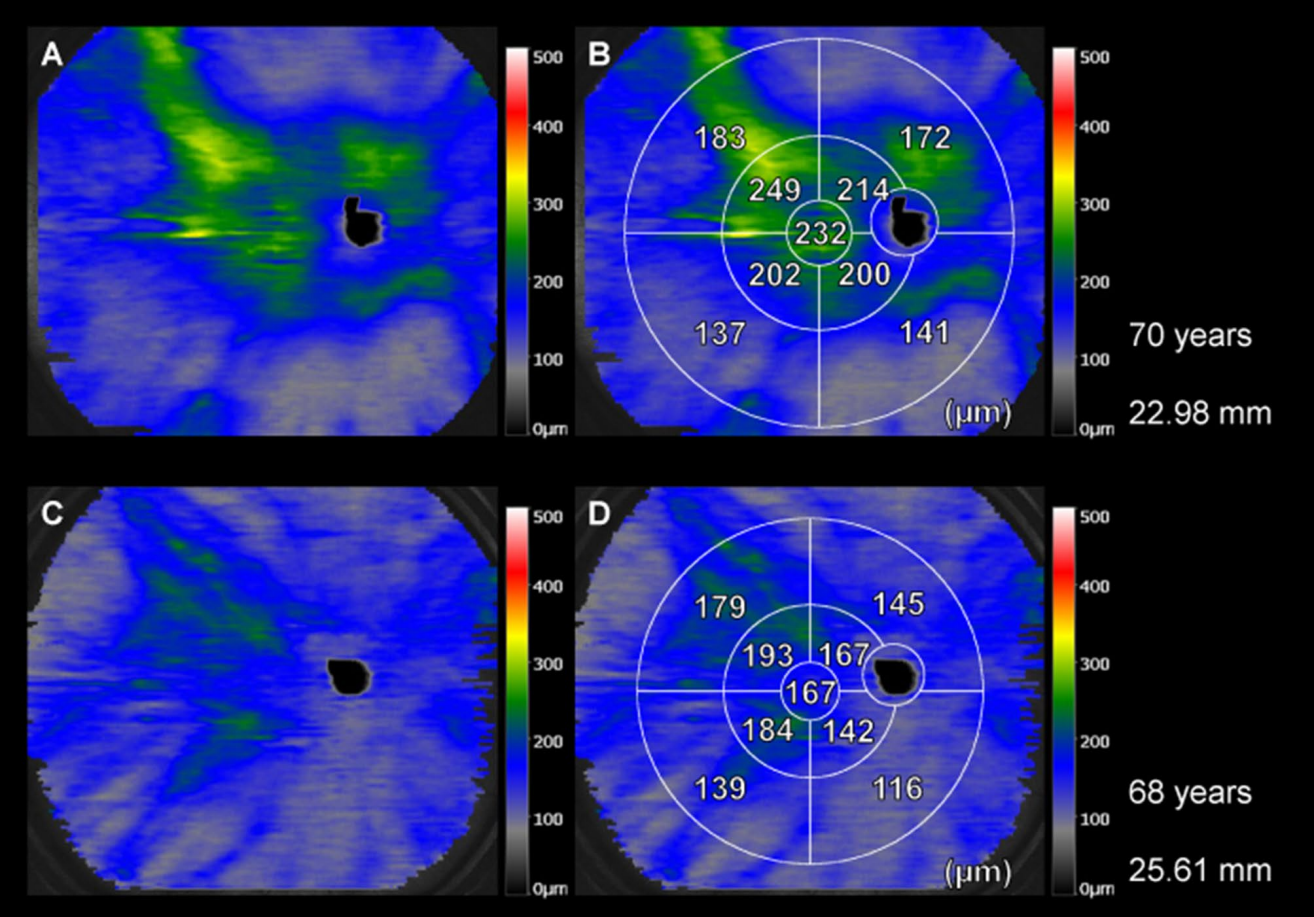
Differences in choroidal thickness according to the axial length. (**A,B**) Widefield (WF) choroidal thickness map of a 70-year-old woman. The axial length (AL) of the eye is 22.98 mm. (**C,D**) WF choroidal thickness map of a 68-year-old man. The AL of the eye is 25.61 mm. These findings suggest that the choroid in all subfields is significantly thinner in eyes with a greater AL (**C,D**) than in eyes with a smaller AL (**A,B**).

We evaluated the effects of age and AL on the WF choroidal thickness (Table 2). Choroidal thickness in all subfields was negatively associated with age (*p* < 0.05; Table 2, Fig. 2, and Supplementary Fig. S2 online), whereas that in the central and inferonasal subfields of the inner and outer rings was negatively associated with AL (*p* = 0.042, 0.034, and 0.022, respectively; Table 2, Fig. 3). The influence of age on the choroidal thickness in these subfields appeared to be stronger than that of AL (standardized β =  − 0.41, − 0.46, and − 0.57 for age and − 0.20, − 0.21, and − 0.21 for AL, respectively; Supplementary Fig. S2 online). We also examined the potential relationship between the spherical equivalent and choroidal thickness across all the studied regions. However, our analysis did not reveal a significant correlation between these variables (*p* > 0.10 for all).

**Table 2:** Multivariable analysis of the association of age and axial length with choroidal thickness in healthy eyes.

Choroidal thickness	Age		Axial length	
	Β	*P*	β	*p*
Central (3 mm)	−2.0	< 0.001	−15.9	0.042
Inner ring (3–9 mm)				
Superotemporal	−2.7	< 0.001	−5.3	0.367
Inferotemporal	−2.6	< 0.001	−11.9	0.068
Superonasal	−1.9	< 0.001	−9.7	0.179
Inferonasal	−1.9	< 0.001	−13.5	0.034
Outer ring (9–18 mm)				
Superotemporal	−1.7	< 0.001	−1.5	0.699
Inferotemporal	−1.8	< 0.001	−7.1	0.075
Superonasal	−1.7	< 0.001	−7.4	0.116
Inferonasal	−1.3	< 0.001	−7.7	0.022

*β* partial regression coefficient.

## Discussion

In the present study, the EDI of WF SS-OCT enabled the generation of posterior pole choroidal thickness maps for healthy Japanese eyes and facilitated the quantitative evaluation of choroidal thickness. The posterior pole choroidal thickness was significantly lower in the periphery than in the central area, in the inferior area than in the superior area, and in the nasal area than in the temporal area. Moreover, choroidal thickness in all the subfields was negatively associated with advanced age, whereas that in the central and inferonasal subfields was negatively associated with AL.

Recent studies have described the morphological features of the choroid in eyes with pathologies using SS-OCT with or without EDI^15,17,20^. The macular choroid has been shown to be thicker in eyes with AMD^6^ and CSC^7^ and thinner in eyes with high myopia^9^. Mori et al. recently examined choroidal thickness in healthy eyes using macular cross-scanning OCT with a length of 6 or 9 mm and showed associations with age, sex, AL, and spherical equivalent^12^. Previous studies have attempted to reveal the characteristics of peripheral choroidal thickness through quantitative evaluation using WF SS-OCT^21,22^. However, most of these studies evaluated only vertical and horizontal B-scans of the macula. The vortex veins in the choroid run obliquely in the superotemporal, inferotemporal, superonasal, and inferonasal quadrants of the posterior pole, and may show characteristic changes on ultra-widefield scanning light ophthalmoscopy images of eyes with CSC and high myopia. Thus, an accurate assessment of such choroidal vascular features using only vertical and horizontal B-scans across the macula would be difficult. Funatsu et al. recently examined the choroidal structure in healthy eyes using 12 long radial scans and reported the WF choroidal thickness features^23^. These results are consistent with the main findings of this study. However, with the imaging protocol used to acquire the radial scans, it may be difficult to capture fine changes in choroidal thickness, particularly at the periphery, because the scanned area is sparser than the more central area (Supplementary Fig. S1 online). In contrast, WF choroidal thickness map analysis performed using the same protocol showed choroidal thickening along the dilated vortex veins in eyes with CSC, which is suggestive of its pathogenesis^15^.

In this study, choroidal thickness was greater in the macular area than in the periphery; the course of the short posterior ciliary arteries (SPCAs) may have played a role in this. SPCAs enter the sclera at a short distance from the optic nerve and run radially toward the equator^24^. The temporal distal SPCAs pierce the sclera to enter the eyeball in the macula and diverge toward the distal portion^24,25^. Choroidal thicknesses in the inferior subfields were lower than those in the superior subfields. A previous study showed that the peripapillary choroidal thickness in the inferior area was significantly lesser than that in the superior area^26–28^, similar to the results of the present study. In the present study, the choroid was thinnest in the inferonasal area, which may be associated with the development of the eye. The optic fissure is located at the inferior aspect of the optic cup and is the last point at which the globe closes^29^.

In addition to the SPCAs, the course of the choroidal veins may affect choroidal thickness. In healthy eyes, the vortex veins extend along the uveal tract, and the vertical watershed between the temporal and nasal vortex veins passes temporal to the optic disc. In contrast, the horizontal watershed between the superior and inferior vortex veins passes between the optic disc and macula^30,31^. The location of the vertical watershed may affect the horizontal choroidal thickness. In the superior area above the horizontal watershed, blood in the superior vortex veins flows against gravity, whereas in the inferior area below the horizontal watershed, blood in the inferior vortex veins flows in the direction of gravity. Thus, the superior vortex veins may be more congested, and the superior choroid may be thicker than the inferior choroid.

In the present study, choroidal thickness in all the subfields was negatively associated with advanced age. This finding is similar to that of previous studies using OCT B-scans^11,12,23,26,27^. Ocular blood flow decreases with advanced age^32^, probably because of an age-related increase in systemic vascular resistance. These changes in the ocular and systemic blood flow may affect age-related decrease in the WF choroidal thickness. In the present study, the choroidal thicknesses in the central and inferonasal subfields were negatively associated with AL. This finding was also consistent with previous findings obtained by OCT B-scans^11,12^. However, in previous reports, morphological changes in the choroid were examined only in the macular area. Thus, the present study may provide additional information on choroidal thickness outside the macular area.

Increasing the AL could potentially lead to the stretching and thinning of the entire choroid. However, in our study, a significant association between AL and choroidal thickness was observed only in the central and inferonasal subfields. Notably, previous studies using dense volumetric scans have provided valuable insights into the choroidal thickness outside the macular region, as well as the impact of vortex veins on the regional choroidal thickness variations across the across the central area (approximately 60°) of the retina in normal eyes^18^.

While these previous studies have provided broad insights, our study further explored the role of vortex veins in the periphery. Our focus has primarily been on the relationship between increased AL and decreased choroidal thickness in the central and inferonasal subfields, which has not been thoroughly explored in the existing literature. Consistent with the findings of Hoseini-Yazdi et al.^18^, we observed a similar association between an increase in AL and a decrease in choroidal thickness in the central and inferonasal areas. Their study evaluated a smaller range of choroidal thickness in 27 eyes, which is consistent with our observations. The strong association between the increased AL and decreased choroidal thickness in these subfields could be attributed to the location of the optic fissure. Our findings, along with the insights gained from the aforementioned studies^18^, contribute to a complex understanding of the various factors that influence choroidal thickness.

This study has several limitations. First, the number of participants was smaller than that in other studies that used a normal database. Second, the choroidal thickness was measured using a viewing angle of 20 (vertical) × 23 (horizontal) mm, which is wider than the viewing angle used in previous studies. However, the areas outside this viewing angle were not evaluated. Third, we did not perform a real-shape correction of the measurement error in choroidal thickness. Real-shape correction may be useful for thickness maps generated from dense radial scans. Instead, we corrected the AL-related magnification using a modified Littmann formula (Bennett procedure) while developing the evaluation grid. Fourth, we did not account for variations in the position of the fovea relative to the optic disc.

Despite these limitations, we examined the two-dimensional features of WF choroidal thickness in healthy eyes of Japanese individuals using thickness map analysis and determined negative associations with advanced age and increased AL. Replication of the current findings is expected in larger cohorts, such as epidemiological consortia.

## Methods

### Participants

This observational study was approved by the Institutional Review Board of the Kyoto University Graduate School of Medicine (Kyoto, Japan) and adhered to the tenets of the Declaration of Helsinki. Written informed consent was obtained from each participant during their first visit before the start of the study.

In line with previous reports^12,23^, we included healthy Japanese participants without any ocular disease other than cataract who were examined at Kyoto University Hospital between October 2021 and September 2022. One eye from each participant was examined for analysis. The exclusion criteria were as follows: history of chorioretinal disease, macular neovascularization, uveitis, scleritis, or corticosteroid use; pregnancy; ocular hypertension (> 21 mmHg) or hypotension (< 5 mmHg); keratoconus; high myopia with a spherical equivalent of <  − 6 diopters; hyperopia >  + 4 diopters; astigmatism >  ± 3 diopters; and poor-quality OCT images (signal strength index < 5) because of eye movements or media opacities. Finally, 127 eyes of 127 participants met the inclusion criteria.

### Choroidal thickness evaluations using EDI of WF SS-OCT

We examined the choroidal structure using SS-OCT (Xephilio OCT-S1, Canon Medical Systems, Japan) with near-infrared illumination at 1010–1110 nm (scanning laser ophthalmoscope, 780 nm) and a scanning speed of 100,000 A-scans per second. The focus spot was set at 30 mm to ensure that the device scanned a large area. No additional lenses or device modifications were required during the image acquisition.

To capture WF choroidal thickness features, we acquired three-dimensional volumetric data using the EDI of SS-OCT with the following parameters: 20 mm (vertical, 128 B-scans) × 23 mm (horizontal, 1024 pixels) with a 5.3-mm scan depth (1396 pixels). For segmentation of the choroid, we set the choroidal thickness as the vertical distance from the Bruch’s membrane to the chorioscleral interface. Segmentation was automatically performed using built-in software supported by artificial intelligence. Because the eyes were free of pathology, automatic segmentation of the inner and outer borders of the choroid was performed correctly.

**Figure 4:**
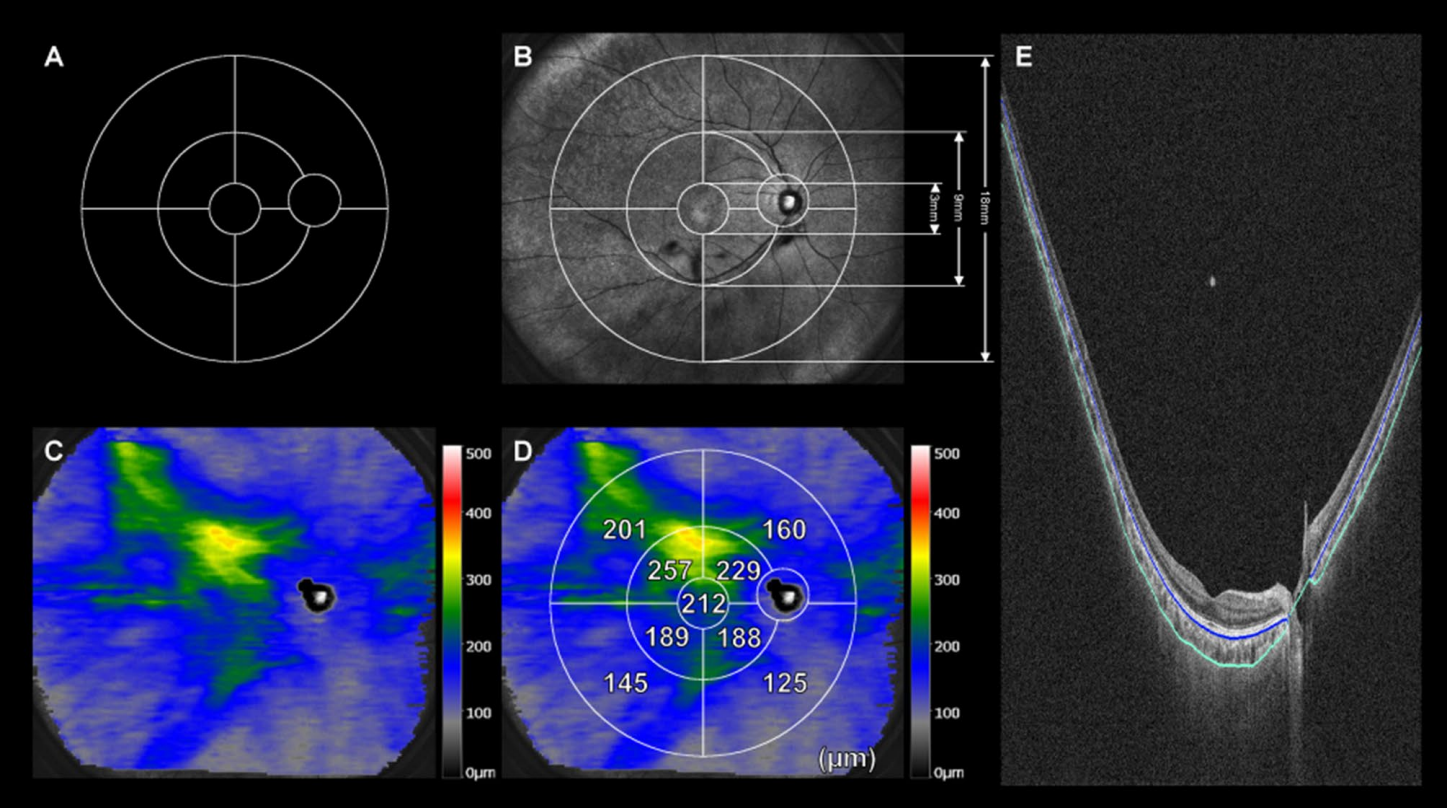
Thickness map analysis of the healthy choroid using enhanced-depth imaging of widefield swept source optical coherence tomography. (**A**) The grid for measuring changes in the wide-field choroidal thickness. It comprises nine subfields divided by three circles with diameters of 3, 9, and 18 mm, respectively, and four lines. Circumferential and zonal areas enclosed by these circles are divided into superotemporal, inferotemporal, superonasal, and inferonasal subfields, with consideration of the arrangement of the vortex veins. (**B**) Infrared scanning laser ophthalmoscopy images overlaid with the measurement grid. (**C**) A choroidal thickness map. (**D**) Choroidal thickness values measured using the grid. The value for each subfield represents the mean choroidal thickness (μm) of the subfield. (**E**) Automatic segmentation of the inner and outer borders of the choroid is performed accurately.

We analyzed choroidal thickness maps using our recently reported method^15^, which is briefly described below. A grid comprising three circles with diameters of 3, 9, and 18 mm was used, and the grid center was always set at the center of the fovea (foveal bulge of the ellipsoid zone^33^) without any rotation (Fig. 4). The inner and outer rings were defined as the fields enclosed between the 3- and 9-mm circles and the 9- and 18-mm circles, respectively. Each ring was divided into superotemporal, inferotemporal, superonasal, and inferonasal quadrants, and measurements were performed for a total of nine subfields, including the central 3-mm ring (Fig. 4).

For choroidal thickness measurements, we unified the measurement range for all participants by correcting for AL-related magnification using the modified Littmann formula (Bennett procedure)^34,35^.

### Statistical analysis

All statistical analyses were performed using JMP pro version 16.2 (SAS Institute Inc., Cary, NC, USA). Values are presented as mean ± standard deviation. Normal distribution of the data was examined using the Shapiro–Wilk test, followed by comparisons between the outer and inner, inferior and superior, and nasal and temporal subfields using the Wilcoxon signed-rank test. To evaluate the association between choroidal thickness and spherical equivalent and AL, respectively, we used different statistical methods. Spearman's rank correlation coefficient was used to assess the relationship between the spherical equivalent and choroidal thickness. Multiple linear regression analysis was used to adjust for each factor when investigating the association of age and AL with choroidal thickness. For all analyses, a *p*-value of < 0.05 was considered to indicate statistical significance.

## Supplementary Information

Supplementary Figure S1.

Supplementary Figure S2.

Supplementary Legends.

## Data Availability

The datasets generated and/or analyzed in the current study are available from the corresponding author upon reasonable request.
