# Supplementary figures and images for: Analysis of widefield choroidal thickness maps of healthy eyes using swept source optical coherence tomography
Source: Sci Rep. 2023 Jul 24;13:11904. doi: 10.1038/s41598-023-38845-9 (PMC10366186; doi:10.1038/s41598-023-38845-9)

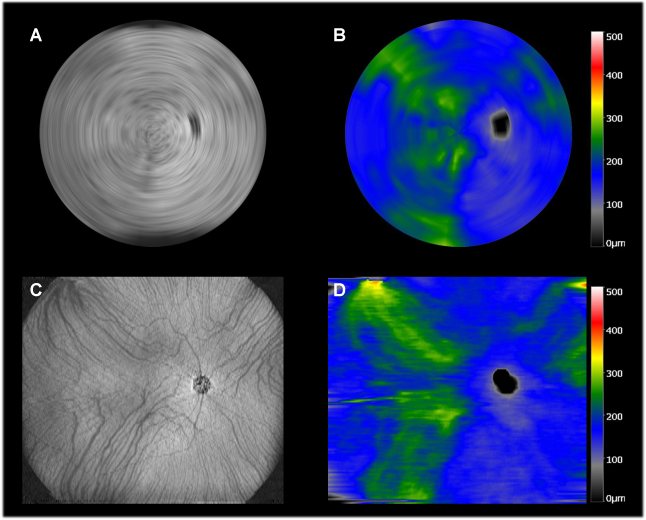

Supplement: Supplementary file 1 — Supplementary Figure S1. [file 41598_2023_38845_MOESM1_ESM.tif]

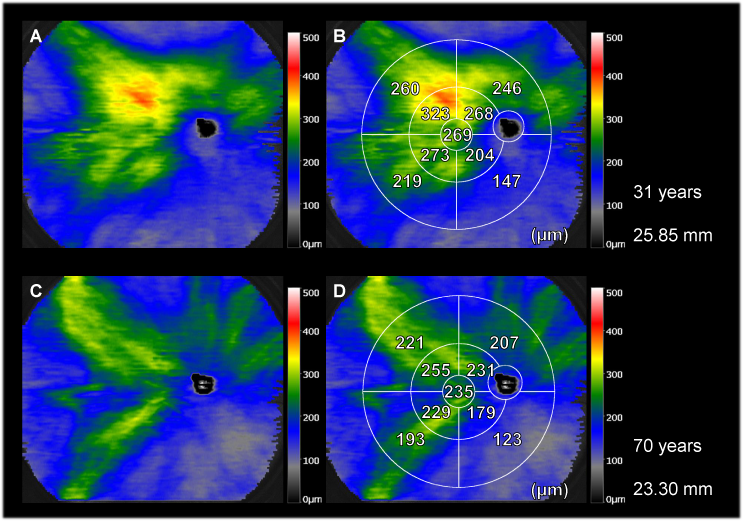

Supplement: Supplementary file 2 — Supplementary Figure S2. [file 41598_2023_38845_MOESM2_ESM.tif]
