# Supplementary material for: Analysis of widefield choroidal thickness maps of healthy eyes using swept source optical coherence tomography
Source: Sci Rep. 2023 Jul 24;13:11904. doi: 10.1038/s41598-023-38845-9 (PMC10366186; doi:10.1038/s41598-023-38845-9)
Supplement: Supplementary file 3 — Supplementary Legends. [file 41598_2023_38845_MOESM3_ESM.docx]

**Supplementary Figure S1. Difference between images created by 12 radial scans and those created by the protocol used in the present study**

A, B. A choroidal en face image (A) and thickness map (B) created from 12 radial scans with a length of 20 mm.

C, D. A choroidal en face image (C) and thickness map (D) created from three-dimensional volume data obtained by enhanced-depth imaging of swept-source optical coherence tomography with a viewing angle of 20 (vertical; 128 B-scans) × 23 (horizontal) mm.

In the images in C and D, the choroidal vessels and thicknesses can be more clearly delineated.

**Supplementary Figure S2. Representative cases with different ages and axial lengths**

**A, B.** A widefield (WF) choroidal thickness map of a 31-year-old woman. The axial length (AL) of the eye is 25.85 mm.

**C, D.** A WF choroidal thickness map of a 70-year-old man. The AL of the eye is 23.30 mm.

Both age and AL are negatively associated with the choroidal thickness in the central subfield (3 mm), inferonasal subfield of the inner ring (3–9 mm), and inferonasal subfield of the outer ring (9–18 mm). However, the influence of age on the choroidal thickness appears to be stronger than that of AL in these subfields (standardized β = −0.41, −0.46, and −0.57 for age and −0.20, −0.21, and −0.21 for AL, respectively).
